# The brain tracks auditory rhythm predictability independent of selective attention

**DOI:** 10.1038/s41598-020-64758-y

**Published:** 2020-05-14

**Authors:** Maja D. Foldal, Alejandro O. Blenkmann, Anaïs Llorens, Robert T. Knight, Anne-Kristin Solbakk, Tor Endestad

**Affiliations:** 1 Department of Psychology, University of Oslo, Oslo, Norway; 2 RITMO Centre for Interdisciplinary Studies in Rhythm, Time and Motion, University of Oslo, Oslo, Norway; 3 Department of Neurosurgery, Oslo University Hospital, Oslo, Norway; 4 Department of Psychology and the Helen Wills Neuroscience Institute, University of California Berkeley, Berkeley, USA; 5 Department of Neuropsychology, Helgeland Hospital, Mosjøen, Norway

**Keywords:** Attention, Perception

## Abstract

The brain responds to violations of expected rhythms, due to extraction- and prediction of the temporal structure in auditory input. Yet, it is unknown how probability of rhythm violations affects the overall rhythm predictability. Another unresolved question is whether predictive processes are independent of attention processes. In this study, EEG was recorded while subjects listened to rhythmic sequences. Predictability was manipulated by changing the stimulus-onset-asynchrony (SOA deviants) for given tones in the rhythm. When SOA deviants were inserted rarely, predictability remained high, whereas predictability was lower with more frequent SOA deviants. Dichotic tone-presentation allowed for independent manipulation of attention, as specific tones of the rhythm were presented to separate ears. Attention was manipulated by instructing subjects to attend to tones in one ear only, while keeping the rhythmic structure of tones constant. The analyses of event-related potentials revealed an attenuated N1 for tones when rhythm predictability was high, while the N1 was enhanced by attention to tones. Bayesian statistics revealed no interaction between predictability and attention. A right-lateralization of attention effects, but not predictability effects, suggested potentially different cortical processes. This is the first study to show that probability of rhythm violation influences rhythm predictability, independent of attention.

## Introduction

Context, behavioral relevance, and predictions provide top-down influences on human perception^1,2^. Bayesian inference, defined as inference that follows rules of probability^3^ is proposed as a core component of sensory perception, and may be used to examine the specific neuronal processes underlying top-down influence^4^. The assumption of Bayesian inference underpinning the interplay between top-down predictions and bottom-up sensory input fits well with the predictive coding theory^5,6^, which is a theoretical computational framework of sensory perception. The main idea is that neural responses do not represent sensory input directly. Instead, they reflect a computed difference between the predicted and the actual sensory input, also known as the *prediction error*. This theory is often used for interpreting effects related to the expectancy or predictability of the sensory input^7^. Here, the terms *expectancy* and *predictability* are used in a statistical manner, assuming that inference follows rules of probability.

A growing body of research suggests that sensory perception and prediction follow rules of probability (for a review, see^7^). One way sensory prediction has been studied is by investigating deviance detection responses, such as the mismatch negativity (MMN) event-related potential (ERP)^8^. The MMN is elicited by violation of several auditory stimulus features such as tone pitch, location, or intensity^9–11^. Relating to rules of probability, it has been shown that the MMN in response to violations, often termed ‘oddballs’ among frequent ‘standards’, are affected by the ratio between the ‘oddballs’ and the ‘standards’. Specifically, the MMN evoked by ‘oddballs’ decreases as the probability of ‘oddballs’ increases^12^. One dimension of sensory predictions that is far less studied is the *time*-dimension, which is surprising given that a crucial part of a prediction would involve prediction of ‘when’ and not only ‘what’. To our knowledge, there are no studies demonstrating that neural responses to specific stimulus-onset-asynchronies (SOAs) are influenced by probability in the same manner as it has been shown for other stimulus features, such as pitch^12^. Hence, the neural computations underlying processing of interval probability are less understood^13^. This is despite several studies reporting that expectancy related to the intervals between stimuli impacts neural processing of sensory input^13,14^. Furthermore, there seems to be a lack of studies investigating more general effects of probability or uncertainty, as the main focus has primarily been on the processing of the unexpected events (i.e., the oddballs). For instance, in rhythmic auditory stimuli, an interval may be considered predictable or ‘standard’ as its timing can be inferred by the rhythmicity in preceding tones. At the same time, occasional violations of the expected rhythm, and the frequency of occurrence of such violations, could render the rhythmic stimuli being perceived as more or less predictable (i.e., less frequent rhythm violations increase the predictability). It remains to be understood how probabilities defined by a given context, such as the probability of rhythm violation, affect neural processing of auditory rhythmic patterns.

Finally, it is not well known how systems involved in extracting rules from auditory input are influenced by attention, another important component of auditory perception. Only a few studies have manipulated predictability and attention independently within the same experiment^15,16^. Yet, there is a lack of studies in which both the predictability regarding stimulus-timing and attention have been manipulated independently. Knowledge regarding the potential interplay between prediction and attention mechanisms is important for understanding auditory perception.

ERP studies have shown that stimulus predictability, including predictability concerning timing between consecutive events, is typically reflected in attenuation of early negativities such as the auditory N1 component (for a review, see^17^). The auditory N1 is associated with early perceptual processing, as it is elicited in response to tone onsets regardless of task demands and is considered an exogenous, stimulus-driven component^18^. However, the amplitude of the N1 is influenced by sustained selective attention and stimulus predictability (for a review, see^19^). This suggests that top-down processes influence auditory responses at early stages of sensory processing. Lange^20^ reported attenuation of the N1 to tones preceded by an isochronous, predictable sound sequence, compared to when the same tone was preceded by sound sequences with random timing. Importantly, the timing of the last tone could be predicted based on the isochronous preceding sequence. Another study from this group investigated whether the effect reflected stimulus predictability, and not merely sequence regularity^21^. The same experimental paradigm was used in this study, but the timing of the last tone was no longer constant. Hence, the exact timing of the last tone could no longer reliably be predicted, and accordingly the auditory N1 was no longer attenuated^21^. Effects of temporal predictability in the N1 time range have also been demonstrated in studies investigating the repetition-suppression effect, an effect that involves attenuation of auditory neural responses with increasing number of prior stimulus repetitions^22^. Specifically, this effect is enhanced if tones are presented with a predictable temporal structure^23^.

However, in this line of research, predictability of event-timing has been manipulated in terms of the regularity in preceding intervals using two specific levels; isochronous versus random. In order to investigate whether the learning of timing-rules in rhythmic stimuli is based on probabilistic inference, a manipulation of probability is required. Furthermore, generalization of prediction mechanisms assumed by computational frameworks, such as predictive coding, to various levels in the processing hierarchy (i.e., predictability based on abstract rules or statistical probabilities) requires an investigation of sensory predictions of increased complexity. For instance, recent findings suggest that an increased level of rhythmic complexity is associated with a reduced MMN in response to rhythm violations^24^. Lumaca, *et al*.^24^ argues that the effect indicates that rhythmic complexity makes it more difficult for the brain to fit a probabilistic model to the stimuli.

Furthermore, it is not known to what degree updating of sensory predictions depends on the behavioral relevance of incoming sensory information. Maneuvering successfully in an environment with an extensive amount of sensory input necessitates directing attention to the most relevant aspects of the environment for effective goal-directed behavior. The ability to prioritize and attend to goal-relevant information while suppressing irrelevant information is referred to as selective attention^19^. Few studies have manipulated predictability and attention independently within the same experiment^15,16^, and even fewer have investigated predictability regarding SOAs specifically^25^. A common topic for these studies is the interaction between attention and deviance processing (rule violations). To our knowledge, only one other study has investigated the interaction between attention and processing of rhythm predictability (regular vs. random). It was found that the N1 was attenuated for tones in a regular compared to a random temporal sequence. Furthermore, the effect was present even when participants engaged in watching a silent video, directing their attention away from the tones^26^. However, it is still unknown whether the difference in N1 amplitude between the regular and random temporal context is driven by extraction of probabilistic rules in the auditory stimuli. Accordingly, it becomes problematic to make assumptions regarding the interplay between attention and specific predictive processes. Experimental designs in which both predictability and attention are manipulated independently, might give additional knowledge regarding assumptions of separate neural networks involved in the effects of predictability and attention. For instance, a right-hemisphere dominance has been suggested for attention processes^27^, while a specialized role of the left hemisphere has been suggested in the processing of rapidly presented stimuli such as musical rhythms^28^. Effects of attention and rhythm predictability on N1 amplitude might therefore have different topographical representations over the scalp.

This current study had two main objectives. The first was to test the idea that the processing of timing rules in rhythmic stimuli is based on probabilistic learning, and how this is reflected in ERP indices of auditory processing, specifically the N1 component. To this aim, participants listened to repeating rhythmic sequences, with a given number of repetitions representing a rhythmic context (experimental blocks). Rhythm *predictability* was defined in terms of the probability of rhythm violations (SOA deviants) for a given rhythmic context. The *high predictability* condition had reduced probability of rhythm violation (less frequent), while the *low predictability* condition had increased probability of rhythm violation (more frequent). This permitted investigation of whether the brain is sensitive to probabilistic information that requires evaluation of a given temporal context. In line with the extant literature, and given that N1 attenuation for regular compared to random temporal structure reflects probabilistic learning of timing rules, we predicted N1 attenuation to tones in the *high predictability* compared to *low predictability* conditions.

The second objective of the current study was to address the interplay between attention and probabilistic learning of interval timing in rhythmic stimuli. Specifically, we tested whether rhythm predictability based on rules derived from probabilities is processed independent of selective attention. To this aim, the auditory rhythm in each block was presented dichotically, with specific tones of the rhythm presented to separate ears. Attention was manipulated by instructing subjects to attend to tones in one ear only. In this way, the rhythmic structure of the tones in both ears combined was identical across experimental blocks, permitting manipulation of auditory selective attention independent from rhythm predictability. As effects of temporal predictability (regular vs random) have been reported when participants do not selectively attend to the tones (i.e., watching a silent video)^26^), we expected to find effects of rhythm predictability independent of attention. Further, assuming different underlying mechanisms for the effect of predictability and attention, we expected the effects of attention and rhythm predictability to be stronger over right- and left hemisphere electrodes, respectively.

## Methods

### Participants

A sample of 34 healthy adult volunteers were recruited for the study. All participants reported having accomplished high-school level education, and 31 reported currently being a student at an institution for higher-level education or having a university- or college degree. All reported normal hearing, no neurological problems, and no cognitive difficulties. Participants also reported not receiving any psychiatric treatment, including no medication for mental illness. None were professional musicians (performing artists, music teachers, or conservatory students). All participants gave written informed consent before participation. The study was approved by the Department of Psychology’s internal research ethics committee (University of Oslo), and was conducted in agreement with the Declaration of Helsinki.

### Stimuli and experimental design

**Figure 1:**
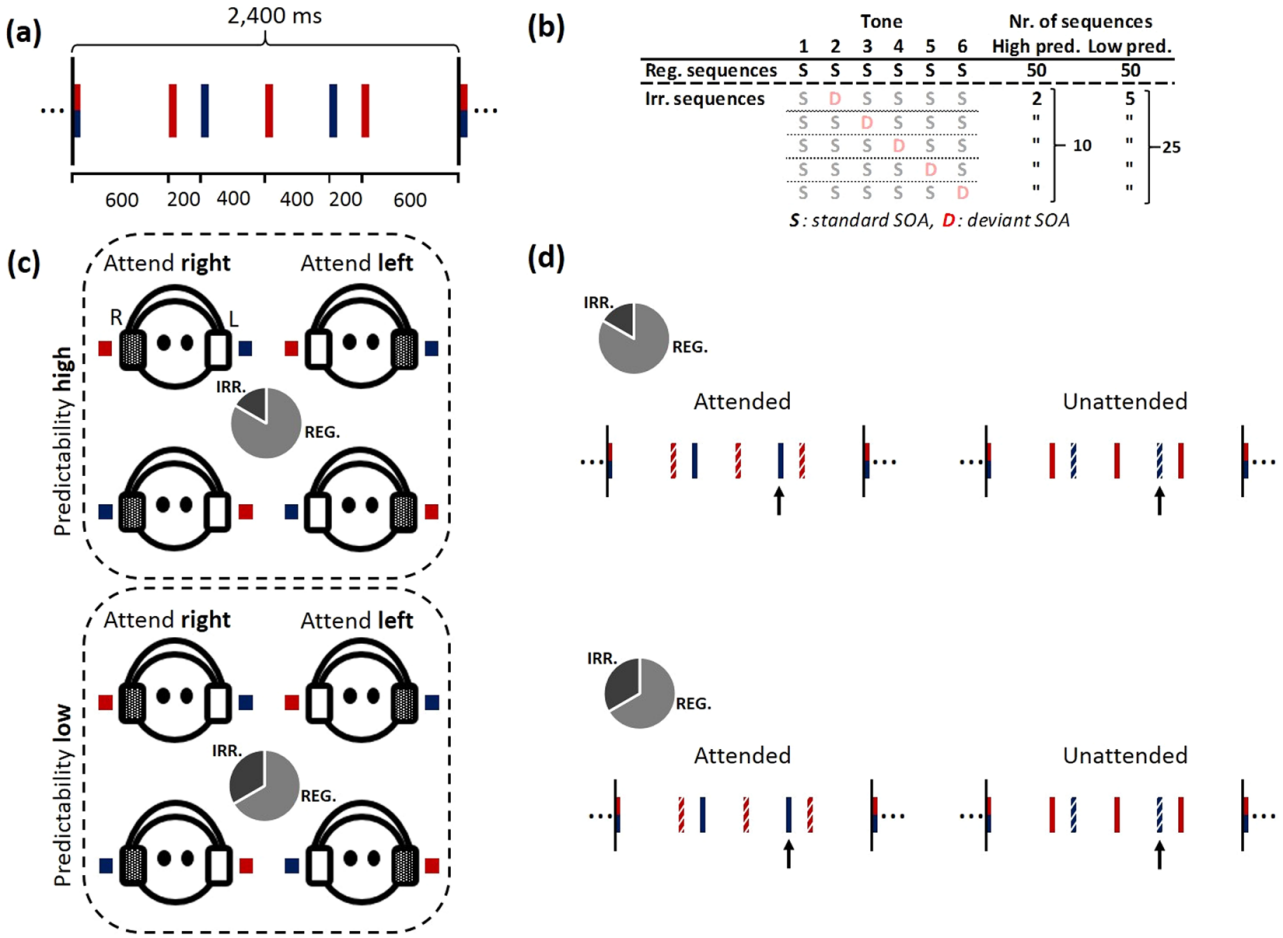
Illustration of the stimuli and experimental design. **(a)** Temporal structure of the 6-tone rhythm sequences. Total sequences duration (above) and SOA between tones (below) are indicated in milliseconds (ms). Colors (blue and red) indicate how the rhythm was dichotically presented. The configuration of which ear (left/right) received which tones (blue/red) was counterbalanced across experimental blocks. **(b)** Schematic illustration of the number of regular (reg.) and irregular (irr.) rhythm sequences per high- and low predictability (pred.) blocks. All possible irregular sequences are illustrated in terms of where in the sequence a deviant (D) SOA was introduced, the number of each irregular sequence, as well as the total number of irregular sequences per high- and low predictability blocks. Irregular sequences are shaded as tones from these sequences were not included in the ERP analysis. Note that the number of events presented in the experiment and the number used for analysis differs. **(c)** Counterbalancing within each participant; attended ear (right vs. left), dichotic configuration (red_right_ – blue_left_ vs. red_left_ – blue_right_), and predictability (high vs. low) resulted in eight experimental blocks. Filled (grey) headphones indicate the attended ear, while empty (white) indicate the unattended ear. **(d)** Rhythm sequences illustrated according to experimental conditions. The black arrows mark the tone of interest (5^th^) used for the N1 analysis. Attention to tones is indicated by solid (attended) or striped (unattended) lines. Top panels represent sequences in *high* predictability blocks, and bottom panels the *low* predictability blocks (the grey circles indicate the proportion of regular and irregular sequences). Left panels illustrate the *attended* condition as the 5^th^ tone appears in the attended ear (solid), while right panels illustrate the *unattended* condition as the tone appears in the unattended ear (striped).

Rhythmic sequences of 6 tones were presented repeatedly. Each sequence had a total duration of 2.4 seconds (SOAs between tones are illustrated in Fig. 1A). For the attention manipulation, the rhythm sequence was presented dichotically, such that specific tones in the sequence (blue color) were presented to one ear, while the other tones (red color) were presented to the opposite ear. The configuration of which ear (left or right) received the specific tones (blue or red) was counterbalanced across experimental blocks. Note that the first tone was presented simultaneously to both ears. Each tone had a duration of 50 ms, with a smooth rise and fall period of 7 ms. We used complex tones consisting of 3 harmonics of a fundamental frequency of 220 Hz. These tone characteristics were identical for all the tones in the rhythm sequence.

Each block consisted of *regular* and *irregular* sequences of tones (Fig. 1B), and eight blocks were presented in total (Fig. 1C). The irregular sequences contained one SOA deviant (−90 ms) in one of the five tones following the first tone. The reason for having SOA manipulations for all tones in the sequence was to make sure participants could not strategically prepare for one single tone in the sequence. Participants were asked to attend to tones in either the left or the right ear during each block, and to respond with a button press to SOA deviants in the attended ear. Participants used their dominant hand and were instructed to respond as quickly and accurately as possible. All blocks contained 50 regular sequences, and rhythm predictability was manipulated by varying the number of irregular sequences (containing a SOA deviant) between blocks. *High predictability* blocks contained 10 irregular sequences (2 SOA deviants for each tone except the first), while *low predictability* blocks had 25 irregular sequences (5 SOA deviants for each tone except the first) (Fig. 1B). The order of regular and irregular sequences within each experimental block was semi-randomized, with the criterion of at least one regular sequence between irregular sequences. Tones were presented dichotically through headphones (i.e., ‘Red’ tones – Left, ‘Blue’ tones – Right). The eight blocks were used to counterbalance the attended ear (left, right), dichotic configuration (red_right_ – blue_left_, red_left_ – blue_right_), and predictability (high, low) (Fig. 1C). After each experimental block, participants received feedback in terms of ‘hit rate’ and ‘false alarm rate’ for that specific block.

### Goldsmith musical sophistication index

As the current study involved rhythmic stimuli, the Goldsmith Musical Sophistication Index (Gold-MSI) questionnaire^29^ was used to assess the participants’ level of musical experience. This allowed us to investigate potential association between the participant’s level of musical experience and the ERP effects. The Gold-MSI is employed as a nuanced measure of musical experience in a non-musician sample, not only involving formal musical training. This self-report inventory has five subscales assessing (1) ‘active musical engagement’, (2) ‘perceptual abilities’, (3) ‘musical training’, (4) ‘singing abilities’, and (5) ‘sophisticated emotional engagement with music’. Finally, a measure of ‘general musical sophistication’ can be computed, based on a selection of items from all five subscales. For the current study, we only used the measure of *general musical sophistication*, in order to have a single measure for each participant that captured both amount of musical training as well as other factors that contribute to a participants level of musical experience. Possible scores ranges from 18 to 126, with higher scores indicating increased level of musical experience.

### Seashore rhythm test

The Seashore Rhythm Test^30,31^ was used to assess the participants’ perception of auditory rhythm. The test requires participants to discriminate between like and unlike pairs of simple musical rhythms. This assessment was used to ensure that all participants included in the analysis fell within the normal range in terms of rhythm perception. Furthermore, the test is considered a useful tool for examining concentration and tracking abilities, meaning that poor performance may reflect deficient rhythm perception and/or tracking abilities^32^. Both rhythm perception abilities (detect rhythm violation) and tracking abilities (sustained selective attention) were crucial in the current study. The range of possible raw scores is from 0 to 30.

### Procedure

**Figure 2:**
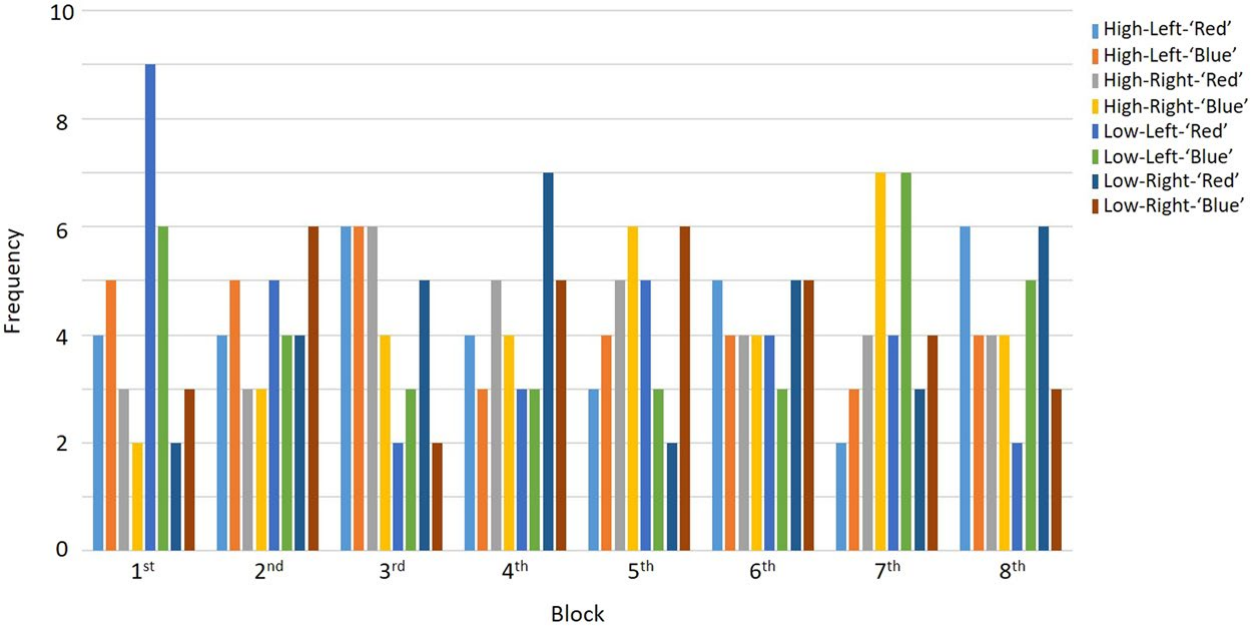
Frequency of each block-type per block-order position (1^st^–8^th^). The color code for each block-type is illustrated in the top right corner, and is given by predictability (high or low), attended ear (left or right), and tones in attended ear (‘red’ or ‘blue’ – see Fig. 1).

At the beginning of the experimental session, participants performed a practice session to get familiar with the stimuli and instructions. The practice involved listening to the stimulus-ear configurations (‘Blue’ Left – ‘Red’ Right, and ‘Red’ Left – ‘Blue’ Right, configurations are also illustrated in Fig. 1C). Each configuration was played twice, once with instruction to tap along with the right-ear tones, and then to the left-ear tones. There was no tapping during the actual experiment. In addition, a short task involving detecting SOA deviants in the attended ear (hits) and ignoring SOA deviants in the unattended ear (false alarms) was conducted to make sure participants understood the nature of the task. After the practice, participants performed eight experimental blocks. At the beginning of each block, eight habituation sequences were played: four monaural (unattended ear silenced) and four binaural. The purpose was to make it easier to direct attention to the instructed side, as well as to familiarize participants with the rhythmic stimuli before introducing the experimental stimuli. There was a break between each block, and the participants initiated the next block when they were ready to continue. The order of the eight experimental blocks was randomized individually for each participant. The frequency of each block-type per block-order position (1^st^ through 8^th^) across all subjects are illustrated in Fig. 2. Each block-type was presented in each block-order position at minimum 2 times and at maximum 9 times.

### Analysis of behavioral data

In order to assess potential association between behavioral performance and electrophysiological effects we computed an overall measure of task performance for each participant. D-prime^33^ was considered an appropriate measure as it captures both the hit rate (H) of the SOA deviants in the attended ear, as well as the false alarm rate (FA) of the SOA deviants in the unattended ear. D-prime was computed for each participant as; z (H) – z (FA).

### EEG-recording and pre-processing

Continuous EEG and electro-oculography (EOG) data were recorded using a BioSemi Active Two 64 Ag-AgCl electrode system (BioSemi, Amsterdam, Netherlands). Electrodes were attached according to the International 10–20 system for electrode placement^34^. Data were sampled at 1024 Hz during online recording. EOG electrodes were positioned above and below the right eye, and lateral to the participant’s left and right eye. Additional external electrodes were positioned on left and right earlobes for later re-referencing.

We used the Fieldtrip toolbox^35^ for Matlab (R2018a, Mathworks Inc., Natick, MA, USA) for offline EEG data processing. Continuous EEG data were filtered with a 0.5 Hz high-pass filter in order to remove slow drifts in the data, as well as possible influence from slow preparatory ERP components (i.e., the contingent negative variation). The data were referenced to earlobes and down-sampled to 512 Hz. Noisy segments of the continuous data and bad channels were identified by visual inspection (i.e., large muscle artifacts). The sample information of the noisy segments was saved for later rejection of epochs overlapping with these segments (see below). Bad channels were removed before running an independent component analysis (ICA). The ICA was used to identify- and then manually remove blinks and horizontal eye movements (ocular components) in the non-epoched data.

The data were segmented into epochs of −500 to 1000 ms relative to the onset of the tones, specifically the 5^th^ tone within the rhythmic sequences (Fig. 1D). Typically, events closer to the end of rhythmic sequences were selected for analyses in previous studies^20,21,24^. Due to the short pre-stimulus interval (200 ms) for the last (6^th^) event in our sequences, we decided to use the 5^th^ event in order to obtain a better baseline for our events of interest (pre-stimulus interval of 400 ms), while still selecting an event towards the end of the sequences. Tones for which the previous sequence contained a SOA deviant were excluded from the analysis, resulting in ~40 trials per each high-predictability block, and ~25 trials per low-predictability block. Epochs were rejected based on previously defined noisy segments in the continuous data if the defined noisy segments overlapped with the first 1000 ms of an epoch (−500 to 500 ms relative to stimulus onset). The mean number of rejected epochs per participant was 4.7. Removed bad channels were re-constructed using spherical spline interpolation to ensure that data from all participants had the same set of channels^36^. Power-line noise was removed by zeroing the components associated with the noise (50 Hz) and its harmonics (100 and 150 Hz) in the discrete Fourier transform of the epoched data. The data were referenced to a common average of all 64 EEG-electrodes, and baseline correction was applied using a 50 ms pre-stimulus (−50 to 0 ms) time-window.

### ERP analysis

Nine fronto-central electrodes were selected for the following analysis of the ERPs, targeting the auditory N1; left hemisphere (F3, FC3, C3), midline (Fz, FCz, Cz), and right hemisphere (F4, FC4, C4). Electrodes were selected based on an expected fronto-central distribution of the auditory N1 component^37^, as well as effects being consistently reported over fronto-central electrodes in other studies manipulating prediction and attention independently within the same experiment^15,16,26^.

#### N1 analysis

For the N1 analysis, ERPs averaged across the midline electrodes (Fz, FCz, Cz) were computed. Only midline electrodes were selected as left- and right ear tones were collapsed for this specific analysis, and we wanted to avoid potential influence of hemispheric difference effects from the lateral electrodes. ERPs to the tones were averaged for each individual as a function of *attention* (attended vs unattended) and *predictability* (high vs low), resulting in four conditions. As left- and right ear tones had been pooled, this resulted in ~80 trials (high predictability) or ~50 trials (low predictability) per condition (the mean number of trials across participants per condition included in the analysis is presented in Table 1). We defined the time-window used to extract N1 mean amplitudes based on the grand average waveform across all participants and all conditions. The two time-points at which the voltage was equal to 50% of the N1 peak value, before and after the N1 peak, served as time-window endpoints^38^. This resulted in a time-window ranging from 82 to 107 ms relative to tone onset, from which the mean N1 amplitude was computed for all four conditions separately.

**Table 1:** Mean number of trials per condition used in the ERP analysis at midline electrodes (across participants, n = 32). The numbers represent the mean after excluding trials preceded by sequences containing SOA deviants, as well as removing trials contaminated by noise in the signal. Note that tones presented to left and right ears were collapsed. Parentheses indicate the standard deviation (SD).

Condition	No. of trials (SD)
Predictability high attended	78.7 (1.5)
Predictability low attended	49.8 (2.1)
Predictability high unattended	79.1 (1.6)
Predictability low unattended	50.4 (1.4)

#### Analysis of hemispheric differences

An analysis of hemisphere effects was included in order to investigate potential hemispheric differences for either attention-related processing or processing of rhythm predictability, as well as for detecting left or right ear advantages in processing the stimuli. It has been shown that enhanced N1 amplitude at contra- compared to ipsilateral electrodes during monaural tone-presentation, is associated with enhanced source-estimated cortical activation in contra- compared to ipsilateral auditory regions^39,40^. Hence, a left-right asymmetry in auditory evoked potentials might reflect different involvement of the left and right cerebral hemispheres. Note that in the current study, hemispheric differences refer to the differences between electrodes positioned over the left and right scalp. The average across the three left hemisphere electrodes (F3, FC3, C3), and the average across the three right hemisphere electrodes (F4, FC4, C4) were used for this analysis.

For investigation of hemispheric differences for attention processes, ERPs were averaged for each individual as a function of stimulated *ear* (left, right), *hemisphere* (left, right), and *attention* (attended, unattended). For investigation of hemispheric differences for rhythm predictability processing, ERPs were averaged for each individual as a function of stimulated *ear* (left, right), *hemisphere* (left, right), and *rhythm predictability* (high, low).

### Statistical analysis

The normality of all data variables were evaluated using the Shapiro-Wilks test and visual inspection of Normal Q-Q plots. Variables with a distribution that differed significantly from a normal distribution according to the Shapiro-Wilks test (*p* > 0.05) were visually inspected using Normal Q-Q plots. For the N1 midline analysis, all variables conformed to assumptions of normality (*p* > 0.05). For the analysis of hemispheric differences one variable in each repeated measures analysis of variance (ANOVA) violated the assumption of normality (*p* < 0.05). However, visual inspection of the Normal Q-Q plots suggested reasonable normal distribution of these variables. Hence, analyses were run using parametric tests.

#### N1 analysis

The mean N1 amplitude averaged across the midline electrodes were analyzed using a repeated measures ANOVA, involving two within-subject factors; *attention* (attended, unattended) and *predictability* (high, low).

Two separate repeated measures ANOVAs were used to analyze hemispheric differences for attention and rhythm predictability effects. The first addressed hemispheric differences for attention effects, and three within-subject factors were included: *attention* (attended, unattended), stimulated *ear* (left, right), and *hemisphere* (left, right). The second addressed hemispheric differences for rhythm predictability effects, and included the three within-subject factors: *rhythm predictability* (high, low), stimulated *ear* (left, right), and h*emisphere* (left, right).

To assess effect strength, omega squared (ω^2^) was computed for all main effects and interaction effects. The ω^2^ is an estimate of the proportion of variance in the dependent variable accounted for by the independent variable. ω^2^-values greater than 0.14 indicate large effects, values between 0.06 and 0.14 suggest medium sized effects, and values between 0.01 and 0.06 are considered small effects^41^.

#### Bayesian statistics

In order to investigate whether our results favored a main effect model of attention and prediction, or an interaction between attention and prediction, we computed Bayes factors (BFs; with default priors (uniform) for repeated measures ANOVA, using JASP free online software, v.0.10.2, https://jasp-stats.org/, University of Amsterdam, Netherlands). The BF is used to compare the probability of two models, in order to determine the plausibility of these models given the data. It is computed as the ratio between the probabilities of the two models. We report the BF as the ratio of the probability of a main effect model and an interaction model:

1 $$BF=\frac{probability\,(main\,effect\,model|data)}{probability\,(interaction\,model|data)}$$

Hence, BF values less than 1 favor the interaction model, while BF values higher than 1 favor the main effect model. We interpreted the BF values according to existing recommendations^42^, considering values between 1 and 3 as anecdotal evidence, and values greater than 3 as substantial evidence in favor of the main effect model.

#### Correlations of N1 amplitude with behavioral performance and musical experience

Finally, we assessed whether the effects of attention and predictability on N1 amplitude were associated with the behavioral measures, specifically d-prime (task performance) and Gold-MSI scores (musical experience). We also assessed whether there was any association between task performance and musical experience. The attention effect was computed as the difference in mean N1 amplitude between unattended and attended tones (unattended minus attended), for each individual participant. For the predictability effect, we computed the difference in mean N1 amplitude between tones in the high and low predictability condition (high minus low), for each individual participant. Two-tailed Pearson’s correlation coefficients were computed to investigate the correlation of attention- and predictability effects with d-prime and/or Gold-MSI scores, and the correlation between task performance and musical experience.

All statistical analyses were performed using JASP (v.0.10.2, https://jasp-stats.org/, University of Amsterdam, Netherlands) free software.

## Results

### Behavioral performance

Two participants were excluded from the analysis. One due to not following instructions properly when performing the experimental task. A second participant was excluded due to Seashore Rhythm test performance below the 5^th^ percentile based on normative data, combined with difficulties understanding and following instructions during the experimental task. The final sample consisted of 32 participants. Group demographics, task performance, and level of musical experience are presented in Table 2. A wide range in the hit rates (0.24 to 0.94) indicated that the task of detecting SOA deviants was demanding. However, the d’prime measure, which takes into account the false alarm rate, suggested that all participants were able to distinguish between the attended and to-be-ignored (unattended) SOA deviants. This was reflected in all participants having a higher proportion of ‘hits’ than ‘false alarms’ (d’prime > 0).

**Table 2:** Group demographics, task performance, and questionnaire data (n = 32). SD = standard deviation (SD).

	Ratio or mean (range)	SD
Gender (females: males)	17:15	
Hand dominance (right: left)	28:4	
Age years, mean (range)	24.1 (19–35)	4.3
**Task performance and musical experience**		
Experimental task		
Hit Rate, mean (range)	0.62 (0.24–0.94)	0.20
False Alarm Rate, mean (range)	0.10 (0.00–0.31)	0.08
d-prime, mean (range)	1.77 (0.42–3.65)	0.82
Seashore Rhythm test, mean raw score (range)	28.3 (25–30)	1.4
Gold-MSI, mean raw score (range)	65.9 (35–107)	17.1

### ERP results

#### N1 effects at midline electrodes

**Figure 3:**
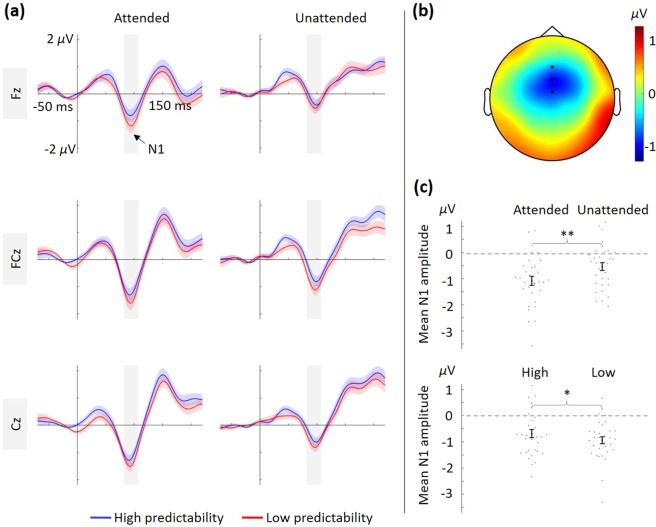
Illustration of results from the N1 analysis at midline electrodes. **(a)** Grand average ERPs at the midline electrodes (Fz, FCz, Cz) for attended (left panel) and unattended (right panel) tones. The high- and low-predictability conditions are illustrated by the blue and red lines, respectively. The shaded area around the lines represents the standard error of the mean. The time-window used for analyzing the mean N1 amplitude (82–107 ms) is shaded in grey. An additional 30 Hz low-pass filter was applied for visualization purposes. **(b)** Topographical representation of the N1 component (82–107 ms) across all task conditions. The three midline electrodes are highlighted (black dots). **(c)** Mean N1 amplitude (82–107 ms) for tones across the midline electrodes (Fz, FCz, and Cz). Plot of the attention effect (top), and the predictability effect (bottom), *p < 0.05, **p < 0.01. Error bars represent the standard error of the mean, and individual data points are plotted (grey dots).

ERP time courses for each condition at each midline electrode are illustrated in Fig. 3A, and the topographical representation of the N1 component across all task conditions is illustrated in Fig. 3B. Analysis of N1 amplitude revealed a main effect of attention, *F*(1,31) = 10.25, *p* = 0.003, *ω*^2^ = 0.076, reflecting enhanced N1 amplitude for attended compared to unattended tones (see Fig. 3C, top). There was also a main effect of predictability, *F*(1,31) = 4.25, *p* = 0.048, *ω*^2^ = 0.017, revealing an attenuated N1 amplitude when predictability was high (see Fig. 3C, bottom). The repeated measures ANOVA showed no significant interaction between attention and predictability on N1 amplitude, *F*(1,31) = 0.06, *p* = 0.808, *ω*^2^ < 0.001. A complementary Bayesian repeated measures ANOVA showed moderate to substantial support^42^ for the main effect model relative to the interaction model, Bayes Factor (BF) = 3.712, indicating no interaction between auditory selective attention and predictability.

#### Hemispheric differences in N1

**Figure 4:**
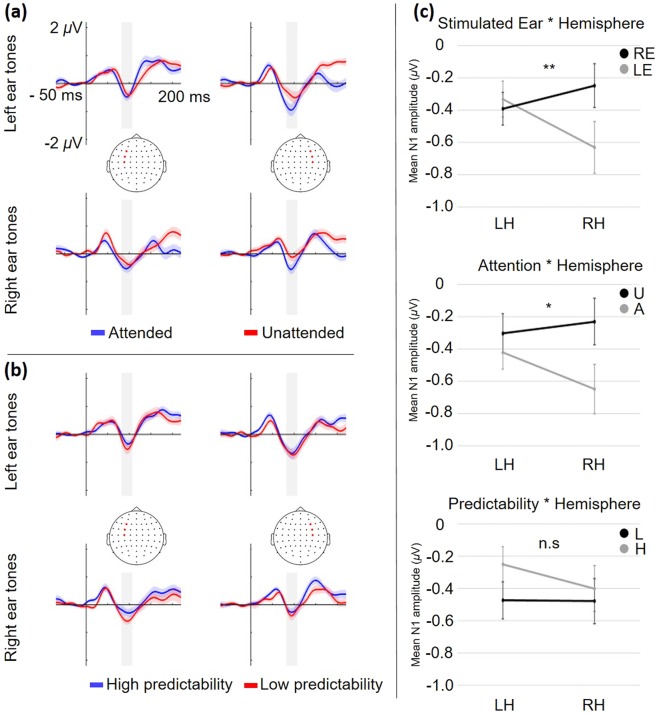
Illustration of results from analysis of hemispheric differences. (**a**) ERP time-courses for attended (blue) and unattended (red) tones. **(b)** ERP time-courses to tones occurring in high predictability- (blue) and low predictability (red) blocks. **(a,b)** Left panels represent the average of left hemisphere electrodes (F3, FC3, C3), and right panels represent the average of right hemisphere electrodes (F4, FC4, C4), highlighted in the head plots with red markers. Top-panels represent left ear tones, and bottom panels right ear tones. The shaded area around ERP time courses represents the standard error of the mean. The time-window used for analyzing the mean N1 amplitude (82–107 ms) is shaded in grey. An additional 30 Hz low-pass filter was applied for visualization purposes. **(c)** Plots of the interaction between hemisphere (LH = left hemisphere, RH = right hemisphere) and the three factors; stimulated ear (top, RE = right ear, LE = left ear), attention (middle, U = unattended, A = attended), and predictability (bottom, L = low pred., H = high pred.). Mean N1 amplitude is plotted for each condition. Error bars represent the standard error of the mean, *p < 0.05, **p < 0.01, n.s = not significant.

Finally, analyses of hemispheric differences for attention- and predictability effects were performed. ERP time-courses for all conditions are presented in Fig. 4A for the attention effects, and Fig. 4B for the predictability effects. Two separate repeated measures ANOVAs were performed, as no significant interaction between attention and predictability was found in the analysis of N1 at midline electrodes.

The repeated measures analysis for the effect of attention on N1 showed a significant main effect of attention, *F*(1,31) = 7.30, *p* = 0.011, *ω*^2^ = 0.033, that was modified by a significant interaction between attention and hemisphere, *F*(1,31) = 4.48, *p* = 0.042, *ω*^2^ = 0.011. Bonferroni-corrected post-hoc comparisons revealed a significant difference only for attended compared to unattended tones over right hemisphere electrodes, *t* = −3.43, *p* = 0.007, reflected by a more negative N1 for the attended tones (Fig. 4C, middle).

The repeated measures analysis for the effect of predictability showed a trend-level main effect of predictability, *F*(1,31) = 3.96, *p* = 0.056, *ω*^2^ = 0.013, which indicated attenuation of N1 amplitude for tones in the high predictability condition. There was no significant interaction between predictability and hemisphere, *F*(1,31) = 1.33, *p* = 0.258, *ω*^2^ = 0.001 (Fig. 4C, bottom).

Neither attention nor predictability interacted significantly with the ear of tone presentation. However, the results revealed a significant main effect of ear, *F*(1,31) = 5.17, *p* = 0.030, *ω*^2^ = 0.013, while the main effect of hemisphere was not significant *F*(1,31) = 0.69, *p* = 0.41, *ω*^2^ < 0.001. Furthermore, there was a significant interaction between ear and hemisphere, *F*(1,31) = 8.05, *p* = 0.008, *ω*^2^ = 0.025. Bonferroni-corrected post-hoc comparisons showed a significant difference only for left- compared to right ear tones over right hemisphere electrodes, *t* = −3.63, *p* = 0.004, reflected in a generally more negative N1 in response to left ear tones across the other experimental conditions (Fig. 4C, top).

#### Correlations of N1 amplitude with behavioral performance and musical experience

Pearson’s correlation coefficients were computed to investigate whether effects of attention and predictability on N1 amplitude were associated with behavioral performance (d-prime) and/or musical experience (Gold-MSI). The N1 attention effect did not correlate significantly with d-prime (r = 0.04, *p* = 0.813) or with the Gold-MSI scores (r = −0.19, *p* = 0.310). Also, the N1 predictability effect did not correlate significantly with d-prime (r = −0.16, *p* = 0.382) or with the Gold-MSI scores (r = −0.04, *p* = 0.838). Finally, there was a significant positive correlation between d’prime and Gold-MSI scores (r = 0.42, *p =* 0.017), showing that participants with higher level of musical experience performed better on the experimental task.

## Discussion

We investigated the effects of rhythm predictability on electrophysiological indices of auditory processing, specifically the N1. Predictability was defined by the probability of rhythm violation (SOA deviants) in experimental blocks consisting of rhythmic tonal stimuli. We also examined whether rhythm predictability was encoded without attentional resources being allocated to the tones. We observed an attenuated auditory N1 to tones in *high predictability* blocks, in which there was a low probability of rhythm violation, while the N1 was enhanced by attention to tones. Importantly, the effect of rhythm predictability occurred independent of spatial selective attention. Furthermore, attention effects were larger over right hemisphere electrodes, while the effect of rhythm predictability did not significantly differ between hemispheres.

The attenuation of the N1 in high predictability blocks is in line with studies showing a reduction in early sensory responses when the timing of stimuli is predictable^20,23^. Predictive coding has been proposed as a possible underlying mechanism^17^. However, in these previous studies predictability was defined primarily by the timing of immediately preceding intervals (i.e., isochronous versus random timing). Lumaca, *et al*.^24^ manipulated the complexity in the regularity of preceding intervals, yet the main focus was on the neural responses to deviance. An assumption of the predictive coding theory is that sensory prediction has a hierarchical organization in the brain, and is accompanied by the premise that inference follows rules of probability^5,43^. It might be problematic to assume a predictive coding mechanism underlying the N1 attenuation for stimuli with regular compared to random timing, as it involves manipulation of neither hierarchical prediction nor probabilities. The current study found attenuation of the N1 in response to tones embedded in rhythmic stimuli when predictability was increased in terms of probabilities. As the timing of immediately preceding stimuli was kept identical across conditions, the effect was not due to differences in the rhythmicity of the preceding intervals themselves. Hence, our results provide support for interpreting N1 attenuation in light of predictive coding, specifically probabilistic inference.

To our knowledge, this is the first study to manipulate rhythm predictability in terms of rhythm violation probability. However, a few studies have shown that MMN responses to pitch deviance are modulated by the probabilistic structure of stimulus pitches^44,45^. Specifically, the MMN in response to tone pitch is increased when tones are embedded within a narrow distribution of tone pitches (low variability) compared with a broad distribution of pitches (high variability). The N1-attenuation in our study is in line with these findings, in which it is argued that the human brain seems capable of tracking the probabilistic structure in auditory stimuli, and not only simple sequence-based rules^44^. Our study reveals that these effects might extend to other dimensions in which stimuli can vary, such as variability regarding SOAs in rhythmic stimuli.

It is still an open question how this effect manifests over time. Rhythm predictability is probably not represented by a constant unique value. Instead, predictions are made continuously based on experience encompassing both shorter and longer time segments. Hence, the observed effect of rhythm predictability on the N1 is a simple representation of the dynamics in the updating of temporal predictions. One potential underlying mechanism could be repetition suppression to the repeating tones, reflecting local adaptation within auditory cortex^46,47^. Suppression of evoked responses to repeating tones has been shown to be modulated by the regularity in the temporal structure with which the tones are presented, at both cortical- and subcortical processing stages^23,48^. In contrast to these studies, in our study tones were presented with the same rhythm structure, and a repetition suppression effect would have to be explained by the time between rhythm violations (SOA deviants), which differs between high and low predictability blocks. Whether SOA deviants in rhythms of varying complexity affect repetition suppression to repeating tones is currently not known. However, the interval between two consecutive tones has been shown to influence adaptation responses, as adaptation is larger for shorter intervals^49^. In this case, we would expect to see larger adaptation (i.e., N1 attenuation) in the low predictability blocks, opposite of our results, as these blocks had a higher number of SOA deviants (shorter intervals). This suggests that other mechanisms might be mediating our effects of rhythm predictability. Furthermore, Garrido, *et al*.^44^, showing that MMN responses are sensitive to the probability distribution of tone pitches, reported that the differences in MMN responses between high and low variability conditions could not solely be explained by local adaptation. It remains to be defined what neural mechanisms are contributing to effects beyond local adaptation effects. Recent studies suggest a specific role of the prefrontal cortex in predicting sensory input based on more abstract and complex rules (i.e., probabilities) in humans^50^ as well as in non-human primates^51^.

Notably, the effect of rhythm predictability was present for attended as well as unattended tones. This was suggested by a non-significant interaction between attention and predictability, as well as by the complementary Bayes factor analysis. The latter showed that a main effect model was more likely than an interaction model. The results are in line with previous studies demonstrating that the human brain responds to unexpected sound stimuli in a variety of different states in which selective attention to the sounds is absent. Some of these studies involved a similar experimental manipulation of selective attention as we employed, specifically the use of an attended and unattended stimulus stream^15,16,25,52^. Others have shown that the brain responds to unexpected sound stimuli even when in a state of reduced consciousness such as under general anesthesia^53,54^, or other causes of coma^55–57^. Garrido *et al*.^58^ addressed the lack of independent manipulations of attention and prediction, as well as pointing to the existence of conflicting findings. They investigated MMN responses to attended and unattended tones, and similar to our results they found no interaction between tone predictability (standard vs. deviant pitch) and attention^58^.

However, this line of research has mainly addressed how rule violation itself is processed independent of selective attention to tones. In the current study, we rather tested the hypothesis that the brain might be sensitive to probabilities in the sensory stimuli independent of attention. Our results concur with research suggesting that the brain is in fact capable of tracking probabilities (i.e., statistical structure) in auditory stimuli, even when the stimuli are task-irrelevant and unattended^44^. A more recent follow-up study indicated that cognitive task-load does not interfere with these processes^45^. At the same time, our study is the first to show that rules of probabilities in the *timing* of auditory stimuli are processed independent of attention, as the previous studies manipulated probability with regard to stimulus pitch^44,45^. Garrido, *et al*.^45^ argued that automatic learning of statistical properties of stimuli allows for perceptual inference in otherwise noisy environments. This renders it possible to detect important changes in the environment while simultaneously engaging in parallel goal-directed behavior. Our results suggest implicit and automatic processing of temporal structure in auditory stimuli related to the probability of event timing in rhythms.

A potential limitation of the current study is that unattended sounds were still part of the temporal structure in the rhythm sequences. In that way they might not be completely task-irrelevant, if one assumes that the brain processes the information from the two streams in an integrated fashion (as one rhythm), and not as separate and independent auditory input streams. If the tones from separate ears were processed as independent streams, an enhancement of the auditory N1 contralateral to the stimulated ear would be expected, due to stronger contralateral than ipsilateral pathways within the auditory sensory system^59^. However, the significant interaction between stimulated ear and hemisphere was driven mainly by a difference over the right hemisphere electrodes, with more negative N1 amplitude to the left ear tones. As the same effect for right ear tones was not present over left hemisphere electrodes, this suggests that left and right ear tones were not processed as independent stimulus streams. Furthermore, the enhanced N1 to left ear tones over right hemisphere electrodes, might reflect a right-hemisphere advantage in processing tonal stimuli^60–62^.

The two predictability conditions might also differ in terms of how one is catching more attention than the other. Possibly, frequent SOA deviants (which occurred for tones delivered to both ears) resulted in more attentional resources being allocated to the unattended ear. In this case, the larger N1 amplitude for *unattended* tones in the low predictability condition could merely reflect increased attention to these tones, and not probabilistic learning. In order to disentangle processes related to sensory prediction and processes related to attention, complementary information regarding the cortical sources of the effects is needed. If the cortical sources differ, this might at least suggest that different processes are involved, and that the effects are not driven by the same attention processes. Interestingly, our result of hemispheric differences in N1 amplitude suggested that attention effects were more strongly lateralized to right hemisphere electrodes. This is in line with previous research suggesting a specialized role of the right hemisphere in auditory attention processes (for a review, see^27^). This specialization has been shown in neuroimaging studies involving healthy participants^63,64^, as well as in studies of participants with acquired brain injury^65,66^. On the other hand, the effects of predictability did not show any significant difference between left- and right-hemisphere electrodes. If any, visual inspection of ERPs would point towards a larger difference between predictability conditions over left hemisphere electrodes. Previous research has suggested left-hemisphere dominance in the processing of rhythm-related aspects of auditory stimuli^28,67^. Our findings suggest that the effects of predictability and attention might result from different cortical processes. However, one should exert caution when making assumptions regarding underlying anatomy based on scalp EEG data alone. Future research investigating the interplay between prediction- and attention processes should find ways to disentangle the specific processes underlying the updating of sensory predictions from attentional processes.

Finally, we found a positive correlation between musical experience and task performance, reflecting that more musically experienced individuals performed better on the task. This result is in agreement with studies showing that musically trained individuals perform better on a variety of rhythm tasks^68,69^. On the other hand, neither performance on the experimental task (d´prime) nor musical experience (Gold-MSI) were associated with the effects of attention and predictability on N1 amplitude. A potential explanation for this is that the ability to detect and respond to the SOA deviants is not directly related to the processing of the probability of SOA deviants. More specifically, one might be able to detect a specific stimulus, even if information regarding its probability of occurrence is absent, as long as one allocates attention to each presented stimulus. This raises interesting questions for future research, i.e., which processes crucial for the perception and understanding of temporal structure in music are influenced by musical experience, and which are not?

In the current study, we have shown that the brain processes rhythm predictability defined by timing probabilities, independent of selective attention. What remains to be understood are the specific neural mechanisms underlying these effects, isolating the mechanisms related to processing of temporal regularities defined by probabilities from those related to local adaptation.

## Data Availability

The ethical approval of the current study does not permit public archiving of the anonymized datasets generated and/or analyzed during the current study. Readers can request access to the datasets supporting claims of the current study by contacting the corresponding author Maja Dyhre Foldal or project manager Tor Endestad (tor.endestad@psykologi.uio.no).
